# Sensorimotor adaptation to sustained lower visual field occlusion during continuous locomotion with and without obstacle negotiation

**DOI:** 10.1007/s00221-025-07111-x

**Published:** 2025-06-04

**Authors:** John G. Buckley, Alan R. De Asha, Brendan T. Barrett, Adam Clansey, Kevin J. Deluzio

**Affiliations:** 1 Department of Biomedical Engineering, Faculty of Engineering and Digital Technologies, University of Bradford, Bradford, UK; 2 School of Optometry and Vision Science, University of Bradford, Bradford, UK; 3 Human Mobility Research Centre, Queen’s University, Kingston, ON Canada

**Keywords:** Visual ex-proprioceptive, Lower visual field (lvf), Adaptive gait, Locomotion, Minimum foot clearance

## Abstract

The importance of having visual feedback of the lower-limb to locomotion control, has typically been examined by intermittently occluding the lower visual field (lvf) in repeated obstacle crossing trials. A consistent finding is that foot clearance increases following lvf occlusion. However, there is some evidence that the increase in clearance diminishes with further repetition. This calls into question the importance of lvf feedback in the control of locomotion. We present two studies investigating how foot clearance is affected as a result of sustained lvf occlusion during continuous locomotion over i) a level surface and ii) the same surface, but involving intermittent obstacle negotiation. In both studies, clearance increased following lvf occlusion but then diminished within a few minutes of continued walking: suggesting that the initial increase may have been an acute but transient response. After four minutes, clearance in level-walking had returned to pre-occlusion levels, whereas for obstacle crossing, clearance remained elevated and showed only a slight lessening over time. These findings provide support for the notion that lvf ex-proprioceptive information is not paramount in the control of the swinging limb/foot during overground gait, but it is customarily used in adaptive gait involving obstacle crossing in determining foot placement before the obstacle and hence clearance over it. We argue that lvf occlusion leads to a more general ‘acute’ perturbation of gait that is not necessarily related to the elimination of visual ex-proprioceptive feedback, and this has implications for the design of laboratory-based studies investigating the role of vision in locomotion.

## General introduction

Research investigating how vision is used to control locomotion has routinely involved adaptive gait tasks, such as obstacle crossing (Patla 1998; Rietdyk and Rhea 2006; Graci et al. 2010; Timmis and Buckley 2012). In these studies, the importance of vision in lower limb control was assessed by investigating the effects of intermittently occluding the lower visual field (lvf) over repeated trials (typically between 3 and 10). Occluding the lvf means that the obstacle is visible up to approximately two walking steps away from the obstacle, but is not visible during the period of crossing, with the result that visual feedback of the feet is eliminated throughout the crossing. Findings from these studies consistently indicate that, following lvf occlusion, gait can still be appropriately adapted to safely negotiate an obstacle. However, toe clearance over the obstacle is increased, with most studies also indicating an increased foot placement distance before the obstacle (Rietdyk and Rhea 2006; Graci et al. 2010; Timmis and Buckley 2012). From such findings, it has been suggested that for the habitual (i.e., unoccluded) situation, central vision is used in a feedforward manner to plan how gait should be adapted to negotiate an obstacle, and then online lvf information of the foot relative to the obstacle (ex-proprioceptive information) is used to control and/or fine-tune foot trajectory and positioning (Patla 1998; Rietdyk and Rhea 2006). It is argued that the increase in toe clearance when lower limb-obstacle visual ex-proprioceptive feedback is unavailable is due to uncertainty regarding the relative locations of the obstacle and the feet, and this leads to margins of safety being increased in order to minimize the risk of tripping.

In a similar vein, it has also been found for the adaptive gait task of stepping onto a raised surface, that toe clearance increases as a result of blurring vision, either diffusely or refractively (Heasley et al. 2004; Vale et al. 2008a, b). In these studies, it was also found that the increased toe clearance diminished with trial repetition, irrespective of whether vision was disrupted or not (Heasley et al. 2004; Vale et al. 2008a, b). Such lessening in foot clearance with trial repetition has also been reported for obstacle crossing involving visual manipulations (Heijnen et al. 2012) or under full or disrupted visual conditions (Graci et al. 2010; Rhea and Rietdyk 2007). It has been suggested that the reduction in toe clearance with trial repetition is due to a drive to conserve energy, since lifting the foot higher requires more energy expenditure (Heasley et al. 2004). The reduction in clearance with repetition may also be a consequence of participants completing the task in an automated/ unconscious manner, because they were required to start their approach from a fixed distance from the obstacle, and then walk up to and over it multiple times. Completing the task in this repeated, predictable manner means that participants would have been less reliant on using visual ex-proprioceptive feedback. It is also possible that the reduction in clearance with repetition is because the initial increase in clearance is partly an ‘acute response’ to having vision perturbed (a response that might occur following any type of visual perturbation), which then abates with time/repetition. If either or both of these scenarios are correct, this would lead to a different interpretation(s) about the importance of online, lower limb-obstacle, visual ex-proprioceptive feedback in the control of adaptive gait. The present study investigates if and how increases in foot clearance as a result of occluding the lvf diminish with time during continuous locomotion (rather than over repeated trials). In an attempt to expand understanding of gait in general, rather than just adaptive gait, we investigate the effects of prolonged lvf occlusion on foot clearance during continuous gait over i) a level, clear surface, and ii) the same level surface but involving intermittent obstacle negotiation.

## Study one. Continuous overground walking with prolonged occlusion of the lower visual field

### Introduction

As the lower limb swings forwards during overground gait, the distal tip of the shoe (the toes) comes into close proximity to the ground; this is known as the minimum toe clearance (MTC). MTC during overground walking is virtually synchronous with peak swing-foot velocity during mid-swing (Winter 1992; Asha and Buckley 2014), which likely contributes to why the risk of tripping is high at the point of MTC (Mills and Barrett 2001). Both the magnitude and variability of MTC are viewed as ‘gait safety’ features (Begg et al. 2007; Mills et al. 2008), whereby increases in magnitude, or reductions in variability, are associated with increased safety. MTC is sensitive to numerous factors. For example, the magnitude of MTC tends to be higher on the non-dominant, compared to the dominant limb (Nagano et al. 2011). MTC increases with walking speed (Schulz 2011; Asha and Buckley 2014), or when the floor surface is irregular (Schulz 2011) or sloped downwards (Khandoker et al. 2010). MTC also reduces for treadmill compared to overground walking (Nagano et al. 2011) or when the floor is sloped upwards (Khandoker et al. 2010). In addition, MTC variability is greater for older compared to young adults (Mills et al. 2008) and is increased when walking both faster and slower than an individual’s ‘customary’ walking speed (Asha 2015).

There is a paucity of research that has investigated whether visual ex-proprioceptive feedback is important for the control of MTC in overground gait. In one of the few studies conducted on this topic, participants repeatedly undertook walking trials under various forms of visual field occlusion (Graci et al. 2009). It was found that occluding the lvf had no significant effect on MTC (mean or variability). However mean MTC (but not variability) did increase if the entire circumferential peripheral visual field was occluded (Graci et al. 2009). It should be noted that all visual field conditions in this previous study were conducted under monocular viewing, where vision in the non-dominant eye was completely occluded throughout. A notable finding from this study was that when lvf information was available (full field vision, upper peripheral occlusion condition), MTC reduced with repetition. This implies that MTC was initially increased in these conditions, even though it was only found to be persistently increased for the circumferential occlusion condition. The initial increase in MTC in all visual field conditions may have been a result of the intermittent occluding of vision, i.e. an acute response to having vision perturbed. However, the finding that MTC only reduced with repetition when lvf information was available would suggest online lower limb-floor ex-proprioceptive information is important to the control of MTC. However, this needs direct examination, which is one of the reasons for the research described here, that aimed to determine if, and in what way, increases in MTC, and/or changes in MTC variability, as a result of occluding the lvf diminish with time during continuous overground locomotion.

### Methods

#### Participants

Eleven healthy young adults (mean (SD) age; 23.0 (2.1) years, height; 1.80 (0.09) m, mass; 80.2 (12.6) kg, 3 females) participated. All stated they were right foot dominant (determined as the limb which the participant would choose to kick a football with). Any participant that habitually used corrective spectacles or contact lenses for walking did so during data collection. All wore their own flat-soled shoes. Prior to data collection, all gave written informed consent. Ethical approval was obtained from the Health Sciences Research Ethics Board at Queen’s University (approval number: 6013096). The procedures used in this study adhere to the tenets of the Declaration of Helsinki.

#### Protocol

**Fig. 1:**
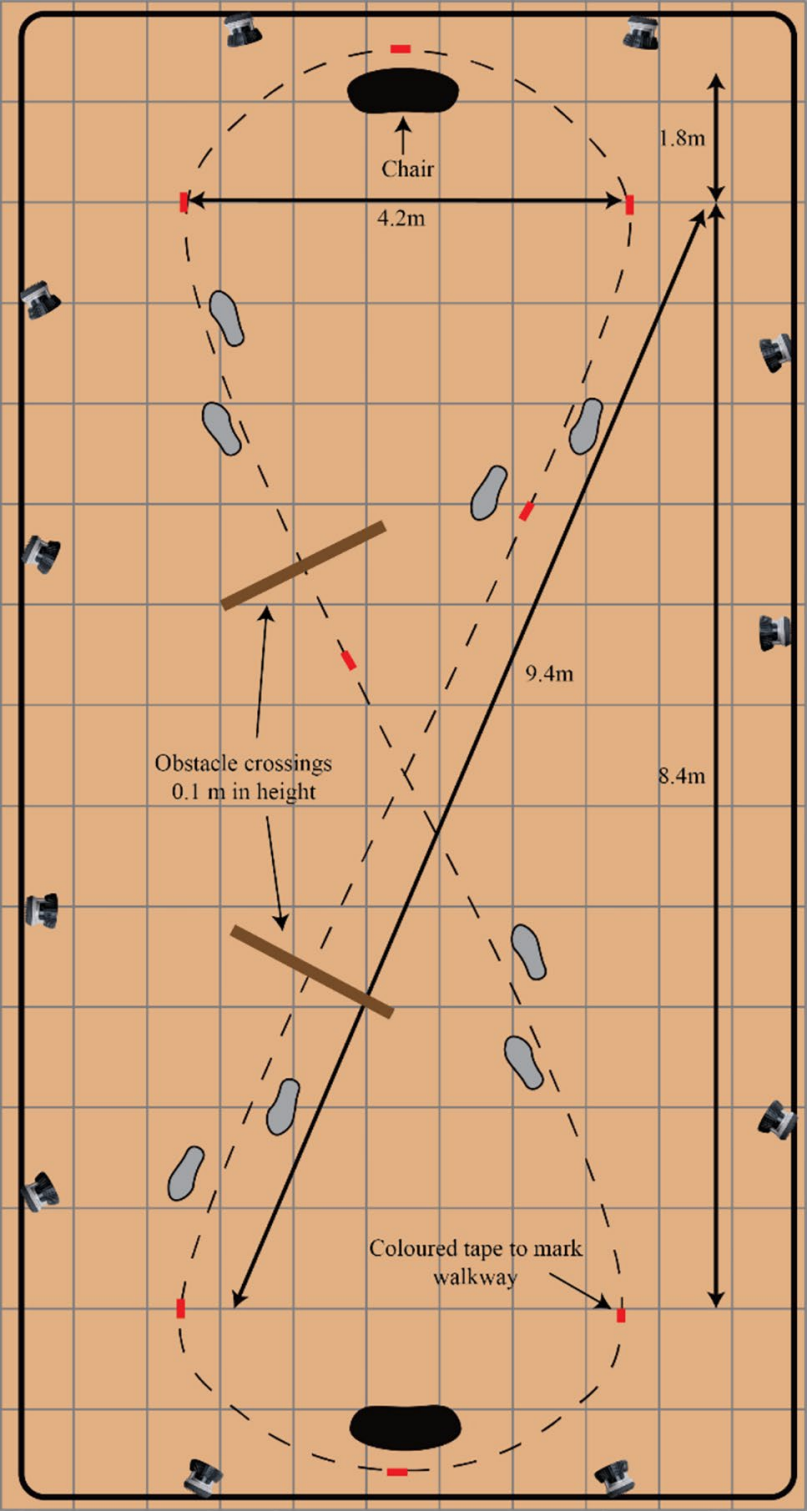
Lab set-up and ‘figure-of-eight’ walking circuit. For Study 1 (Overground Walking) the obstacles were not present. For study 2 (Obstacle Crossing), the obstacles (indicated by the brown lines) were intermittently placed for the duration of the first, fifth and tenth minutes of continuous walking

A ‘figure-of-eight’ walking pathway was marked out on the laboratory floor using small pieces of coloured sticking tape placed on the floor, spaced approximately every 2 m (Fig. 1). The ‘figure-of-eight’ pathway (arranged within a rectangle measuring 16 m by 7 m) provided two ‘straights’, each approximately 9.5 m long. Each participant was instructed to walk along the pathway “at the speed they would normally walk” for two, 10 min periods, with a gap of at least five minutes between each period. No other instructions were given. During both periods, participants wore standard safety-type goggles. For one period, they wore a pair of goggles that were covered with adhesive tape on the lower half of the googles, with the tape’s upper edge level with the participant’s lower eyelids so as to occlude the lvf. Tape was also placed on the sides of the lower half and on the bottom edge of the goggles to eliminate visual feedback in the lvf from around the edges of the goggles. As the tape, over the googles was in line with the lower eyelids, the eyes would have been angled slightly downwards by approximately 10 degrees, meaning 80 degrees of the lower visual field would have been occluded with the head in a neutral position. However, when walking the head will be titled downward (flexed) by approximately 20–30 degrees (Marigold and Patla 2008), which would mean only 50–60 degrees of the lvf would be occluded. This meant that participants (group average eye level height of 1.72 m) would not have been able to see anything in the lvf that was closer than 2-3 m away in front of them. Thus, they would have also been able to see their lower limbs, but they had unobstructed vision of the environment and floor from beyond approximately 2–3 m in front of them. In the other period, participants wore a pair of clear goggles. The order of vision condition (full field/unoccluded, lvf occlusion) was counterbalanced across participants.

#### Data acquisition and processing

A head-band with five reflective markers was worn by each participant. In addition, four reflective markers were placed on the upper surface of the shoe of each foot, distal to the ‘crease’ indicative of the metatarsal-phalangeal joint line. The latter markers were used to track a virtual landmark at the antero-inferior edge of the shoe (shoe-tip). This shoe-tip virtual marker was defined using a digital pointer (C Motion, Germantown, MD, USA). Kinematic data were recorded at 100 Hz using 11 infrared cameras (Qualisys, Gothenburg, Sweden). Data were labelled within Qualisys Track Manager software (Qualisys Gothenburg, Sweden) and then exported in C3D format to Visual3D (C Motion, Germantown, MD, USA), where all subsequent processing took place.

#### Data analysis

Marker trajectory data were interpolated and low-pass filtered with a 6 Hz cut-off. For each participant, the following parameters were determined: average walking speed, along with the MTC for each walking step completed, over the two ‘straights’ of the pathway (~ 12 steps on each straight). MTC was defined as the minimum vertical distance between the floor and the shoe-tip during mid-swing. Walking speed was defined as the average forwards velocity of the head segment’s centre of mass. We knew that MTC would not be normally distributed (Begg et al. 2007; Mills et al. 2008; Graci et al. 2009), thus the median and interquartile range were used as measures of the central tendency and of the variability. MTC parameters were calculated separately for the dominant and non-dominant limbs. Median values were not normally distributed (skewness = 2.2, kurtosis = 5.0), and thus they were log_10_ transformed, which brought skewness and kurtosis within acceptable levels (−0.19 and 1.4, respectively). All parameters were recorded as the average for each minute in the two 10 min periods.

#### Statistical analyses

All parameters were analysed using repeated measures analysis of variance (ANOVA). MTC median-log_10_ (median) and MTC IQR (variability) were compared with visual field (full, lvf occluded), limb (dominant, non-dominant) and time (first through to last minute) as factors. Walking speed was compared with visual field and time as factors. Post hoc analyses were made using Tukey HSD tests. The alpha level was set at 0.05. All inferential statistical analyses were undertaken using Statistica for Windows version 8.0 (StatSoft, Inc., Tulsa, OK, USA).

#### Results

**Fig. 2:**
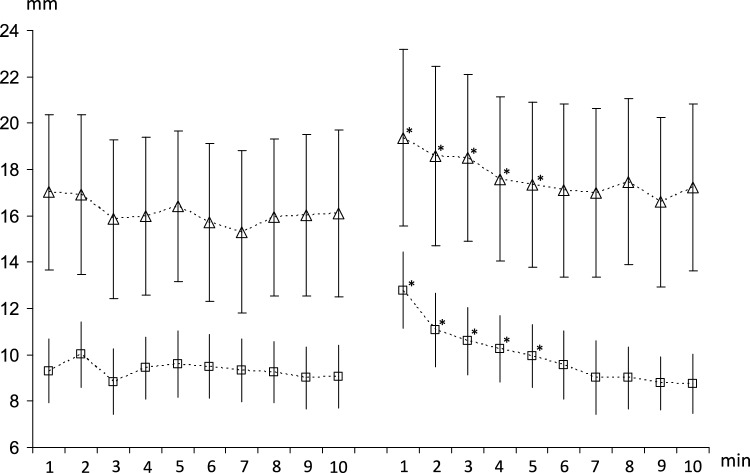
Group average (± SE) MTC median for consecutive, one-minute periods (minutes 1 to 10) for the dominant (lower panels) and non-dominant (upper panels) limbs for full field (left-hand panels) and lvf occluded (right-hand panels) conditions. The symbol *, indicates significantly different to full field condition (*p* < 0.05)

**Fig. 3:**
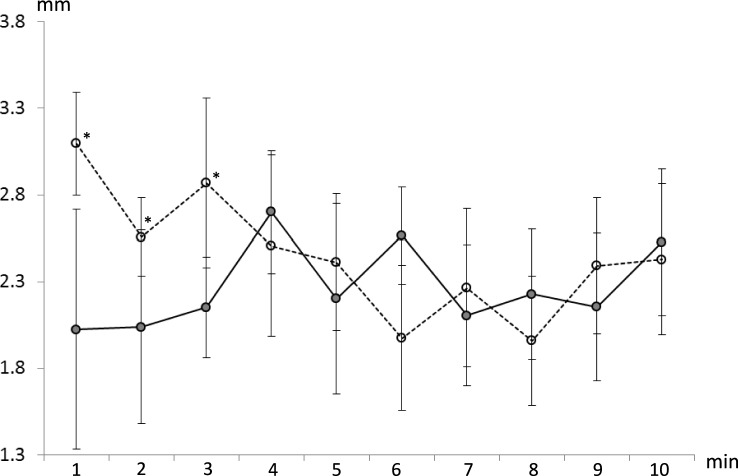
Group average (± SE) MTC inter-quartile range for consecutive one-minute periods (minutes 1 to 10) for full field (solid line) and lvf occluded (dashed line) conditions. Symbol *, indicates significantly different to full field condition (*p* < 0.05)

There were no significant main effects of visual field (*p* = 0.10) or limb (*p* = 0.10) on MTC median, though at each time point MTC median was higher on the non-dominant compared to the dominant limb. There was a significant effect of time (*p* < 0.001) and a significant time-by-visual field interaction effect (*p* = 0.003) on MTC median. Post hoc analysis indicated that MTC median was greater for the lvf occluded compared to full field condition for minutes 1 through 5 (*p* < 0.004, Fig. 2). This highlights that MTC median was increased during the initial period of lvf occlusion but then reduced back to full field levels after approximately five minutes. There were no main effects of visual field, limb or time on MTC variability (*p* > 0.46), but there were significant interaction effects between visual field and limb (*p* = 0.030), and visual field occlusion and time (*p* = 0.024). Post hoc analysis indicated that there was no difference in MTC variability across time in the full field condition, whereas in the lvf occluded condition MTC variability was higher during the first minute compared to minutes 7 through to 10 (*p* < 0.029, Fig. 3).

In addition, there was no difference in MTC variability (average across times) between limbs when the lvf was occluded or between visual field conditions for the dominant limb (p = 0.99). However, for the non-dominant limb MTC variability (average across times) was lower for the full field compared to lvf occluded condition (*p* = 0.024). There were no significant effects of visual field occlusion (*p* = 0.20), time (*p* = 0.41) or interaction effects (*p* = 0.93) on walking speed, although group mean walking speed was consistently lower when the lvf was occluded compared to the full field condition (mean walking speed: Full field, 1.43 m/s; lvf occluded, 1.40 m/s).

## Discussion

The initial abrupt increase in MTC median and MTC variability when the lvf was occluded, highlights that the elimination of lower limb-floor visual ex-proprioceptive feedback had an initial impact on participants’ gait. However, walking speed was not significantly affected by lvf occlusion. The lack of a limb-by-visual field effect on MTC median suggests that this initial impact affected both limbs, though the significant limb-by-visual field effect on MTC variability suggests the effect was slightly greater for the non-dominant compared to dominant limb. Under lvf occluded conditions, MTC median and MTC variability began to return towards pre-occlusion levels relatively quickly (i.e. within one minute), and returned to unoccluded levels after approximately four minutes of walking.

It is possible that participants adapted to the lvf occlusion by adjusting their head angle downwards so as to obtain visual feedback from the floor immediately in front of them from the upper visual field (i.e., by viewing above the occluded portion of the goggles). Therefore, we retrospectively compared the sagittal plane head angle between visual field conditions (i.e. with and without lvf occlusion) and across time intervals using a repeated measures ANOVA, but found no significant differences (*p* > 0.6). This indicates that participants made no attempt to gain lower limb-floor visual ex-proprioceptive feedback by adjusting the head angle to view above the occluded portion of the goggles they were wearing.

A possible explanation for the abrupt increase in MTC mean and variability during the initial period of lvf occlusion is that it was an acute response to the visual field perturbation. Such ‘acute’ responses, which have been referred to as ‘first trial responses’, have been reported for various experimental conditions involving unexpected perturbation of balance or induced slips (Brady et al. 2000; Marigold et al. 2003; Oude Nijhuis et al. 2009; Nanhoe-Mahabier et al. 2012). However, in such studies there is an unexpected ‘physical’ (mechanical) perturbation, that if not responded to would lead to loss of balance or a slip. In the present study the lvf was occluded prior to participants starting their walk around the figure of eight path, and thus the perturbation was not unexpected. Nevertheless, the occlusion of the lvf caused an acute increase in MTC median and variability, and although both quickly began to return to unperturbed levels it took approximately 4–5 min of continuous walking to fully return to unoccluded levels. This highlights that participants were initially more cautious when the lvf was occluded but then quickly began to adapt to it.

One possible means of adapting to lvf occlusion could involve a switch to utilising other types of sensory information to control the swinging limb/foot, e.g. lower-limb proprioception in combination with visual ex-proprioceptive feedback from the upper visual field. Such a switch would be consistent with sensory re-weighting and/or cue-combination models, as applied to the control of upright posture (Peterka 2002) or prehension (Greenwald and Knill 2009). That is, whenever cues that are typically available in a given situation (e.g. lower limb-floor visual ex-proprioceptive feedback during overground gait) become unavailable and/or degraded, the system attaches increased weight to the next most reliable/useful cues. Hence in the present study, participants may have become more reliant on limb proprioceptive feedback under the lvf occluded condition, and the initial increase in MTC variability may be indicative of the CNS adjusting to this sensory pathway. The fact that MTC median and variability did return to unperturbed levels suggests that online lower limb-floor visual ex-proprioceptive information is not paramount to the control of the swinging foot during overground gait. This is consistent with why participants made no attempt to gain such information from the upper visual field by further tilting their heads downwards.

Although MTC median was not significantly different between the dominant and non-dominant limbs, at each time point, the MTC median was higher on the non-dominant compared to the dominant limb (Fig. 2). Limb dominance has previously been shown to have an influence on foot clearance, with greater foot clearance seen for the non-dominant compared to dominant limb with a similar difference between the limbs as that found in the present study (Nagano et al. 2011). This difference may be related to ‘Handedness’ or laterality, whereby fine-motor control is better for the preferred (dominant) limb. However, the specific mechanisms underlying limb dominance effects are still not fully understood (Sadeghi et al. 2000).

## Study two. Intermittent obstacle crossing during continuous walking with prolonged lower visual field occlusion

### Introduction

Obstacle crossing is a discrete event, which only occurs sporadically during everyday walking. Successful and safe obstacle negotiation involves appropriate placement of the feet before and after the obstacle, and adequate clearance over it (Patla 1998). Vision is known to be important for this adaptive gait task: for review see (Marigold 2008). Feedforward information gained via central vision is used to plan the appropriate gait adaptations (to enable appropriate foot placements) and determine the obstacle’s height and other important characteristics (exterioceptive information) (Rietdyk and Rhea 2006; Rhea and Rietdyk 2007; Timmis and Buckley 2012; Heijnen et al. 2014). Then, feedback from the lvf is used online to update (‘fine tune’) foot placement relative to, and foot trajectory over, the obstacle (ex-proprioceptive information) (Rietdyk and Rhea 2006; Rhea and Rietdyk 2007; Timmis and Buckley 2012). Hence, when the lvf is occluded and visual ex-proprioceptive information is unavailable, foot placement distance from an obstacle, and the clearance over it, is increased. Such findings have led to the conclusion that lower limb-floor visual ex-proprioceptive information is habitually used online in controlling the foot during obstacle crossing (Patla 1998; Rhea and Rietdyk 2007). Based on the findings from Study One above, it is also possible that the increase in toe clearances that occur following lvf occlusion is, at least in part, an acute response to the visual perturbation. If this is the case, such a response would diminish with time/repetition following a period of adaptation after the introduction of lvf occlusion.

The aim of the present study was to determine if and how increases in foot placement distance before an obstacle, and the clearance over it, that arise as a result of occluding the lvf, diminish over time. We asked participants to walk around the same ‘figure of eight’ walking pathway as used in Study One, but this time an obstacle was placed in their travel path for the duration of the first, fifth and tenth minutes of walking; participants crossed over the obstacle twice during each of these 1 min periods. Participants again completed two 10 min walking bouts, one under full-field conditions and the other with lvf occlusion. If under lvf occluded conditions, the increases in toe clearance over the obstacle returned back to unperturbed levels within approximately 5 min of walking, this would indicate that lower limb-obstacle visual ex-proprioceptive feedback is not paramount to the control of the swing limb/foot, and thus that the induced initial increase in clearance was an acute response to having vision perturbed. However, if toe clearance remained higher than the unperturbed level for the duration of the 10 min walking trial, even if it initially partly diminished towards unperturbed levels, then this would suggest that lower limb-obstacle visual ex-proprioceptive feedback is paramount for the control of the swing limb/foot during adaptive gait involving obstacle crossing.

### Methods

Eighteen healthy young adults (mean (SD) age; 21.5 (2.5) years, height; 1.77 (0.09) m, mass; 72.6 (13.2) kg, 8 females) participated. None had taken part in Study One. Note, for each study (Study One and Study Two), recruitment numbers were based on convenience samples; no sample size calculations were undertaken a priori. The disparity in sample size between studies is therefore chance occurrence. As in Study One, prior to data collection all participants gave written, informed consent. Ethical approval was obtained from the Health Sciences Research Ethics Board at Queen’s University (approval number: 6013096). The procedures used in this study adhere to the tenets of the Declaration of Helsinki.

### Protocol and data acquisition and processing

The protocol and data collection approach were identical to Study One, except that participants negotiated a 10 cm high, 3 cm deep and 2 m wide wooden obstacle that was intermittently placed in their travel path during the ten minutes of continuous walking around the figure-of-eight walking circuit (Fig. 1). Participants were instructed to walk around the figure-of-eight circuit at their customary walking speed and, whenever an obstacle was present, to step over it. No other instruction was given. The obstacle was placed approximately 1 m past halfway along one of the 9.5 m straights in the figure-of-eight circuit for the duration of the first, fifth and tenth minutes of continuous walking. Each participant crossed the obstacle twice each time during the one minute period when the obstacle was present. Thus, they crossed the obstacle six times in each visual field condition. For each visual field condition, kinematic data were recorded for the periods of obstacle crossing (i.e. from approximately two-to-three steps before the obstacle, and for one step after crossing the obstacle).

### Data analysis

For each participant, the following parameters were determined: lead-limb vertical toe clearance (VTC), lead and trail foot placement before the obstacle, lead foot placement after the obstacle, and approach speed to the obstacle. VTC was defined as the vertical distance between the top of the obstacle and the ‘shoe-tip’ when it was directly above the obstacle. Foot placements were defined as the horizontal distance between each ‘shoe tip’ (during ground contact) and the obstacle. Approach speed was defined as the average forwards velocity of the head segment’s centre of mass for the period of ‘straight-line walking’ during the approach to the obstacle up to the instant of VTC (i.e. over approximately 4–5 steps).

### Statistical analyses

Data were normally distributed and hence were compared using repeated measures ANOVAs with visual field (full, lvf-occluded), time period (first, fifth and tenth minute) and crossing occurrence within each time period (first and second crossing) as factors. Post hoc analyses were made using Tukey HSD tests. The alpha level was set at 0.05. All statistical analyses were undertaken using Statistica for Windows version 8.0 (StatSoft, Inc., Tulsa, OK, USA).

### Results

**Fig. 4:**
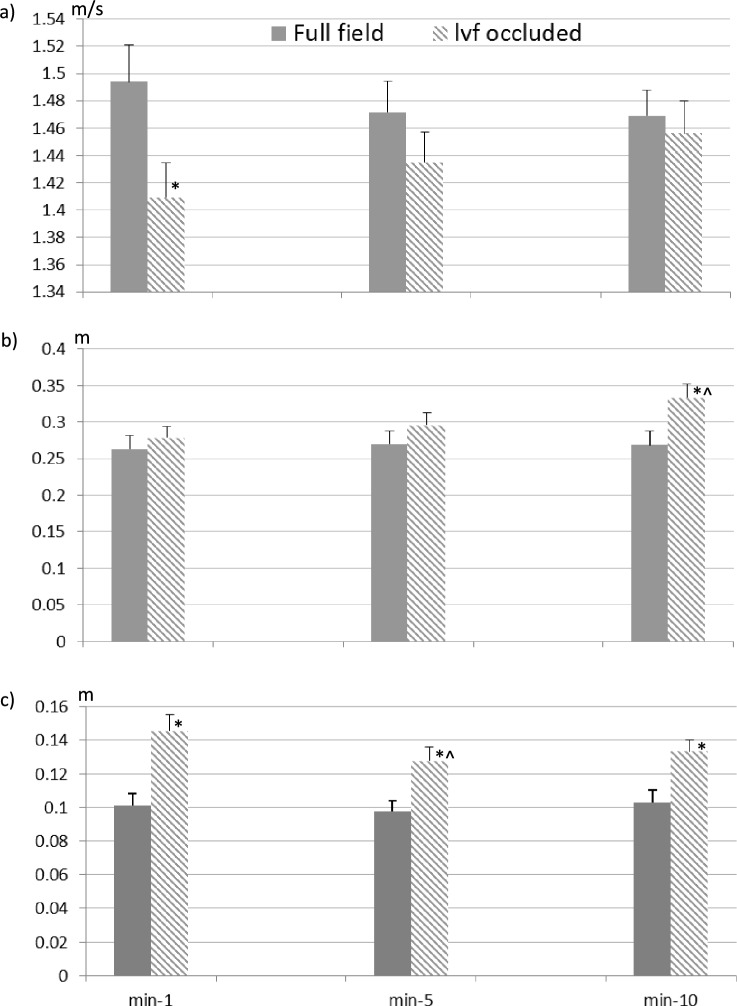
Group mean (+ SE) obstacle crossing a) approach speed, b) trail-limb foot placement before obstacle, and c) vertical toe clearance for minutes 1, 5 and 10 during the ten minutes of continuous walking under full field and lvf occluded conditions. Values are average of the two crossing occurrences that occurred within each one-minute period. The symbol *, indicates a significant difference relative to the full-field condition (*p* < 0.005), and the symbol ^ indicates a significant difference relative to minute-1 (*p* = 0.028)

Under each visual condition the obstacle was repeatedly crossed without contacting it, with either the leading or the trailing foot. Lead-limb VTC was significantly affected by visual field (*p* = 0.002), time period (*p* = 0.011), but not by crossing occurrence (*p* = 0.86). There were no interactions between terms (*p* ≥ 0.14). VTC was higher, on average, when the lvf was occluded (13.6 cm) compared to full field condition (10.0 cm). Across both visual field conditions, VTC became reduced with time period, with post-hoc analyses indicating a significant reduction in toe clearance between the first and fifth minutes (by an average of ~ 1.1 cm, *p* = 0.025), but no difference between the fifth and ten minutes (*p* = 0.30, Fig. 4c). Further inspection of the data indicated that the reduction in clearance between the first and fifth minutes was greater for the lvf occluded (~ 1.79 cm, *p* = 0.028), compared to full field condition (~ 0.42 cm, *p* = 0.097).

Approach speed was significantly affected by visual field condition (*p* = 0.005), but not by time period (*p* = 0.82) or crossing occurrence (*p* = 0.14). However, there was a significant time period-by-crossing occurrence interaction (*p* ≤ 0.0013). The average approach speed was lower when the lvf was occluded (1.43 m/s) compared to full-field vision (1.48 m/s; Fig. 4a), and was greater, across visual conditions, for the first crossing occurrence compared to the second during the first minute of walking. However, there were no speed differences between crossing occurrences during the fifth or tenth minutes. There was also a visual field-by-time period interaction trend on approach speed (*p* = 0.08), which indicated that the approach speed reduced slightly from the first (1.5 m/s) to the tenth (1.47 m/s) minute under full field conditions, whereas it increased from the first (1.42 m/s) to the tenth (1.46 m/s) under lvf occluded conditions. This trend indicated that although approach speed was reduced for the lvf occluded compared to full field condition across all time periods, the reduction in walking speed was more obvious for the first minute of walking compared to that in minutes 5 and 10 (Fig. 4a). Lead-foot placement (i.e., penultimate step) before (average: 1.074 m full field, 1.057 m lvf occluded) and placement after (average: 0.318 m full field, 0.323 m lvf occluded) the obstacle were unaffected by visual field, time period, or crossing occurrence (*p* > 0.24). Trail-limb foot placement (i.e., final step) before the obstacle, however, was affected by visual field condition (*p* = 0.010) and by time period (*p* = 0.042), but was unaffected by crossing occurrence (*p* = 0.22), and there were no interactions between terms (*p* > 0.21).

Trail-limb foot placement distance was greater when the lvf was occluded (30.26 cm) compared to the full-field condition (26.72 cm), and for both visual field conditions it increased with time period, with post-hoc analyses indicating no significant increases between the first and fifth, or fifth and tenth, minutes (*p* > 0.36); however, there was an increase between the first and tenth minutes (by an average of ~ 3.0 cm, *p* = 0.033; Fig. 4b). Further inspection of the data indicated that this increase in placement distance with time period was mainly related to increases for the lvf occluded condition, with little change in placement distance across time periods in the full field condition.

## Discussion

Occlusion of the lvf resulted in a reduction in approach speed and increases in trail-foot distance before the obstacle, and the lead-foot clearance over it. These results suggest that, following elimination of limb-obstacle visual ex-proprioceptive information, participants’ gait became more cautious and/or uncertain and, as a consequence, the margins of safety were increased. However, by the fifth minute of lvf occlusion, the difference in approach speed between visual field conditions became non-significant. Although lead-limb VTC reduced slightly from its initial increase following lvf occlusion, it was still significantly higher compared to full-field conditions, and it remained so for the duration of the 10 min walking trial. These results suggest that there was an initial acute response to the visual perturbation, which partly diminished after approximately 4–5 min of walking. This is evidenced by the increase in walking speed, which returned to the unperturbed level, and by the increase in VTC, which partly returned to the unperturbed level. However, the fact that VTC remained significantly elevated throughout the 10 min of walking in the lvf occluded condition suggests that a lack of lower limb visual ex-proprioceptive information caused uncertainty, and as a result margins of safety were increased throughout. The implication is that visual feedback of the foot relative to the obstacle (ex-proprioceptive information) is important and habitually used, in controlling the swinging foot during obstacle negotiation.

Although the trail-foot distance before the obstacle was greater for the lvf occluded compared to full-field condition across all time periods, it was only significantly greater at minute 10, with no significant differences in foot placement distance between visual field conditions at minutes one and five. This finding is contrary to previous research which has shown that placement distance relative to the obstacle instantly increases following lvf occlusion (Patla 1998; Rietdyk and Rhea 2006; Graci et al. 2010; Timmis and Buckley 2012). This contrary finding may be related to differences in the respective experimental protocols. In previous reports, participants completed repeated trials involving 3–8 m of straight line walking, starting from a fixed location and in which participants were typically instructed to cross the obstacle placed within their travel path with the same limb each time (Patla 1998), (Rietdyk and Rhea 2006), (Graci et al. 2010), (Timmis and Buckley 2012). By contrast, in the current study there was no instruction regarding which limb to lead with when crossing the obstacle, and crossing occurred during continuous locomotion. As well as trail-foot placement distance increasing with time period under lvf occlusion, the approach speed also increased (trend only) with time period in the lvf occluded condition. A retrospective analysis indicated a weak but significant correlation between approach speed and trail-foot placement distance (*r* = 0.19, *p* = 0.004), and this was the case even if only the data for the lvf occluded condition, or only the full field condition, was included. This provides further support for the notion that foot placement distance before an obstacle is a key determinant of safe/successful obstacle negotiation.

The increases in VTC following lvf occlusion were found to lessen from minute-1 to minute-5 but there were no further reductions evident at minute-10. The only other variable found to change between minute-1 and minute-5 in the lvf occluded condition was walking speed, which was seen to increase. A retrospective analysis indicated that VTC was significantly negatively correlated with walking speed (*r* = −0.15, *p* = 0.03). However, including only data for the lvf occluded condition showed no such correlation (*r* = −0.07, *p* = 0.46). This suggests that although VTC may vary with walking speed under habitual full field conditions, there is no such relationship when the lvf is occluded. This provides further support for the notion that lvf ex-proprioceptive information is indeed important for the control of the foot during obstacle crossing; i.e. it is not simply a consequence of walking speed.

## General discussion

We have presented the results of two studies designed to examine whether the initial impact of lvf occlusion persists over time. The results of Study One (involving overground walking, without obstacles) indicated that following lvf occlusion, there was an initial abrupt increase in MTC median and MTC variability, but both measures began to quickly diminish and they both returned to full-field levels after approximately four minutes of walking. We interpret these findings as indicating that online lower limb-floor visual ex-proprioceptive information is not paramount for the control of the swinging foot during overground gait. In Study Two (involving obstacle crossing), participants walked around the same ‘figure-of-eight’ pathway as used in Study One and they did so under the same visual field conditions; however, this time they were asked to cross an obstacle that was intermittently placed in their travel path. The results indicated that although the initial increase in VTC following lvf occlusion diminished after 4–5 min of walking, VTC remained significantly elevated compared to full-field conditions for the duration of the 10 min walking period. We interpret these findings as indicating that lower limb-obstacle visual ex-proprioceptive feedback is essential for safe/successful obstacle crossing. We interpret the initial abrupt increase in foot clearance in both studies following lvf occlusion, as an acute transient response to the visual perturbation.

It has previously been shown that feedforward visual exproprioceptive information regarding final foot-placement (Timmis and Buckley 2012) and the height of an obstacle (Heijnen et al. 2014) is key in determining the lead-limb’s foot trajectory over an obstacle and hence foot-obstacle clearance margins (Timmis and Buckley 2012), (Heijnen et al. 2014). For the obstacle crossing trials in the present study, feedforward visual exproprioceptive information regarding final foot placement would have been unavailable when the lvf was occluded, and this would have subsequently caused uncertainty regarding the trajectory (and hence clearance) of the lead limb’s foot over the obstacle. In contrast, for overground walking the proprioceptive information about the swinging foot would be constant across the repetitive walking steps and thus there would be no requirement for feedforward visual exproprioceptive information regarding the height of the swinging foot above the ground. The relative increased importance of visual exproprioceptive information for obstacle crossing compared overground walking may explain why the initial increase in VTC during obstacle crossing did not reduce back to full field levels, whereas the initial increase in MTC during overground walking following occlusion did revert back to full field levels.

Another factor to consider is the number of times the obstacle was crossed. As the obstacle was placed in participants’ travel path for the duration of only the first, fifth, and tenth minutes, by the end of the 10 min participants only crossed the obstacle 6 times. In contrast, during the 10 min on the clear walkway, participants would have completed over 1000 steps. Thus, the participants would have had many less steps to complete sensory re-weighting in the obstacle crossing study compared to overground walking study. Of course, in the obstacle crossing study participants would have become familiarised with the lvf occlusion during walking around the figure of eight path irrespective of whether the obstacle was negotiated or not. Even so, the relatively fewer obstacle crossings completed under lvf occluded conditions compared to the multiple steps completed during overground walking under lvf occluded conditions, may partly explain why the initial increase in foot clearance following occlusion of the lvf did not reduce back to full field levels for obstacle crossing, contrary to what took place for the overground walking scenario. It is possible that had the obstacle been present continuously, so that they crossed it on every lap around the figure-of-eight pathway, we may have found different effects of time or repetition. The reason we only placed the obstacle in participants’ travel path intermittently was because this is more reflective of how obstacles are encountered in everyday life, i.e. they are only encountered occasionally. Furthermore, we knew from Study One that any ‘acute’ response to the visual perturbation should have subsided after 4–5 min of continuous walking. Thus, if VTC was going to fall back to pre-perturbation levels, it should have done so by minute 5. Our findings showed that this was not the case, and VTC remained elevated throughout the 10 min of walking. Future work could perhaps determine whether increases in obstacle crossing VTC as a result of occluding the lvf diminish in the same way when an obstacle is present continuously (encountered on every lap) versus being present only intermittently.

Following lvf occlusion, walking speed during overground gait (Study One: full field mean, 1.43 m/s) was reduced (trend only), and the approach speed to the obstacle (Study Two: full field mean, 1.48 m/s) also significantly reduced. Interestingly, overground walking speed remained reduced under lvf occluded conditions throughout, whilst obstacle-crossing approach speed increased with time and was close to full field levels by the end of the 10 min of walking. The somewhat contrasting effects on walking speed across the two studies cannot be related to the chosen walking speed, as there was no difference in the group mean walking speeds between the studies (*p* = 0.4). However, the range of chosen walking speeds across participants was much greater for overground walking (1.16 to 1.82 m/s) compared to obstacle crossing (1.29 to 1.67 m/s). The reduced variation in chosen walking speed across participants in the obstacle crossing study may be due to obstacle crossing imposing mechanical constraints on freely chosen walking speed. It is also possible that the increased variation in chosen overground walking speed in participants in Study One partly explains why the changes in walking speed following lvf occlusion did not reach statistical significance.

When crossing obstacles (3 to 15 cm high), mean foot clearance is typically around 10–12 cm (Patla 1998; Rietdyk and Rhea 2006; Graci et al. 2010). This corresponds to the clearance we found in Study Two. Hence, any lessening of toe clearance following the initial increase resulting from the lvf being occluded, could occur within relatively large margins of safety. When walking overground on a flat and level surface, median MTC is normally less than 2 cm with toe clearance variability as much as 50% of this Begg et al. (2007), Asha and Buckley (2014), Graci et al. (2009), Mills and Barrett (2001), and again this corresponds to the clearance and variability we found in Study One. Hence, any increase in MTC as a result of occluding the lvf would be less likely to fall back towards pre-perturbation levels because the margins of safety are so small. Thus, the fact that the initial increase in MTC following occlusion of the lvf reduced back to full field levels for overground walking, but not for obstacle crossing (where margins of safety are greater), provides further support for the notion that online lvf ex-proprioceptive information (i.e., visual information of the foot relative to the ground) is not customarily used in overground walking on a clear and level surface, but it is essential to the control of the foot during the adaptive gait task of obstacle crossing.

The findings presented here may have important implications for the design of lab-based studies, particularly regarding the number of repeated trials to complete and/or the duration of continuous walking to use. If Study One had only collected data for one or two minutes of continuous walking, it is likely that a different and erroneous conclusion, that lvf ex-proprioceptive information is essential to the control of the swinging foot during overground locomotion, would have been drawn. By collecting data for 10 continuous minutes, this allowed us to reach the conclusion that online lvf ex-proprioceptive information is not in fact customarily used during overground locomotion to control the swinging foot, but the opposite is true during the adaptive gait task of crossing obstacles.

There are a number of limitations in the present study. A lack of segmental kinematic data meant we were unable to determine how foot/toe clearance was modulated in the two studies. However, our aim was to establish if, and in what way, sensorimotor adaptation occurred following occlusion of the lvf, rather than describe the movement strategies involved in such adaptations. As such we restricted the number of retro-reflective markers to a minimum, which allowed participants to wear their everyday, comfortable clothes, and we hoped this would ensure that their performance was as natural as possible within the laboratory environment. This straight-forward approach also meant that we could only assess changes in toe clearance, rather than determine whether or not the ‘toe’ was indeed the part of the foot that was closest to the ground or obstacle. Again, as our aim was to establish what sensorimotor adaptations occurred following occlusion, we believe the approach of looking just at toe clearance was acceptable in satisfying our aims.

## Conclusions

These results suggest that the increases in foot clearance that occur following lvf occlusion are at least partly an acute response to visual perturbation. This response maybe one that occurs following any type of experimental sensory perturbation. In addition, by collecting data for a period long enough to allow such an ‘acute response’ to abate, the present study provide support for the notion that lower-limb visual ex-proprioceptive information is not paramount for the control of the swinging foot in gait over a level surface but, as others have shown, it is customarily used in adaptive gait involving obstacle negotiation in determining the foot placement before the obstacle and the trajectory (and thus clearance) of the swinging foot over the obstacle. However, the comparatively fewer obstacle crossings completed under lvf occluded conditions in comparison to overground steps completed, mean that future work is needed to confirm this notion. The results of the present studies indicate that the duration of visual manipulations used needs to be considered when designing studies featuring them, because the magnitude of their impact may reduce considerably over relatively short time periods following their introduction.

## Data Availability

No datasets were generated or analysed during the current study.
